# Assessing Body Image in Multiple Sclerosis: Adaptation and Validation of the Modified Body Image Scale

**DOI:** 10.1002/brb3.71796

**Published:** 2026-10-16

**Authors:** Erica Grange, Alessia Susini, Giampaolo Brichetto, Federica Di Antonio, Jessica Podda

**Affiliations:** ^1^ NeuroBRITE Research Center Italian Multiple Sclerosis Foundation (FISM) Genoa Italy; ^2^ Department of Informatics, Bioengineering, Robotics and Systems Engineering (DIBRIS) University of Genoa Genoa Italy; ^3^ AISM Rehabilitation Service Italian Multiple Sclerosis Society (AISM) Genoa Italy; ^4^ Department of Experimental Medicine (DIMES) University of Genoa Genoa Italy

**Keywords:** body image, cross‐population adaptation, multiple sclerosis, PROMs, psychometric properties

## Abstract

**Background:**

Multiple sclerosis (MS) is frequently associated with physical, cognitive, and emotional changes, including functional loss, which may significantly affect body image (BI) perception. However, a BI questionnaire specifically adapted for use with people with MS (PwMS) is currently lacking. This study aims to adapt and validate the original Italian version of the Body Image Scale (BIS) for the MS population and to examine its psychometric properties.

**Methods:**

A three‐phase adaptation process was conducted, involving a panel of MS experts and PwMS to ensure coherence with MS clinical characteristics, as well as item relevance and clarity. The psychometric properties of the modified BIS (mBIS) were evaluated, including content validity, construct validity, internal consistency, test–retest reliability, and minimal detectable change (MDC).

**Results:**

The modified version of the BIS was established. A total of 208 PwMS (mean age (SD): 50.9 (14.6) years; females: 144; mean self‐reported EDSS (SD): 4.0 (1.96)) completed an online survey. The one‐factor structure was confirmed (CFI = 0.977, TLI = 0.968, SRMR = 0.059), with high internal consistency (McDonald's *ω* = 0.963; Cronbach's *α* = 0.942); and good test–retest reliability (ICC = 0.88). The mBIS showed a moderate correlation with anxiety (*r* = 0.52), a high correlation with depression (*r* = 0.67), and a low negative correlation with interoceptive awareness (*r* = −0.24) (all *p* < 0.001). The MDC was 6.73 and SEM for total score was 2.88. The mean (SD) mBIS score was 12.9 (9.0).

**Conclusions:**

This study supports the validity and reliability of the mBIS as a tool for assessing BI in PwMS. The findings highlight the relevance of BI concerns in MS and their potential role in psychological well‐being.

## Graphical abstract

This study supports the validity and reliability of the modified Body Image Scale (mBIS) as a tool for assessing body image in PwMS, highlighting the relevance of body image concerns in MS and their potential role in psychological well‐being.

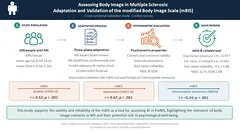

## 1 Introduction

Body image (BI), known as the way an individual perceives, thinks, or feels about their own body (Grogan 2021), is fundamental for awareness, development, and maintenance of individual psychological identity. Each time modifications occur in the body, the psychological perception of the body must be modified as well and adapted in the context of changing physical (Di Cara et al. 2019). Multiple sclerosis (MS) frequently involves the development of physical, cognitive, and emotional changes, including loss of function, that are likely to affect BI in this way.

The sense of owning and perceiving one's body depends not only on afferent bodily signals, but also on the multisensory integration of interoceptive, exteroceptive, and proprioceptive information with pre‐existing body templates (Tsakiris et al. 2007). This integration relies, at least in part, on distinct neural substrates, which have been conceptualized as comprising three distinct components: the experience of owning a body (body ownership), the experience of being localized within one's body at a given position in space (self‐location), and the experience of perceiving the environment from the perspective of one's body (first‐person perspective) (Serino et al. 2013). This can be selectively altered through multisensory body‐related illusions, such as the rubber hand illusion paradigm, in which the synchronous visuo‐tactile stimulation of a visible fake hand and the participant's own hidden hand induces the illusory feeling that the fake hand belongs to the self (Tsakiris et al. 2007). Research indicates that specific multisensory brain areas are intrinsically connected to the immune system. Consequently, immune‐mediated diseases, such as MS, may disrupt multisensory integration, thereby affecting the representation of BI (Costantini 2014). When the neural networks supporting this multisensory interplay are compromised, as may occur in immune‐mediated conditions, the resulting distorted or unstable body representation may therefore translate into an altered BI. Consistent with this, the first study that explored BI in MS was published in 1985 (Halligan and Reznikoff 1985), showing that alterations in BI appeared soon after diagnosis.

It has been shown that in PwMS, increased dissatisfaction with BI affects personal value and sense of self, leading to lower self‐esteem, higher depression, and more frequent sexual dysfunction (Di Cara et al. 2019). In addition, a recent study (Lo Buono, Bonanno, et al. 2023) demonstrated that the degree of disability was an important risk factor for the development of greater BI‐related distress. Because of the strong links between BI alteration and poor health outcomes, it is important for clinicians to be able to evaluate this with solid measurement tool.

To the best of our knowledge, no BI questionnaire has been specifically developed for people with neurological disorders. BI has nevertheless been investigated in several neurological populations using instruments originally developed for other clinical or general populations. A recent systematic review (McCormack et al. 2025) identified up to 14 different questionnaires that have been used to assess distinct aspects of the BI, such as satisfaction with physical appearance, interoceptive awareness, or perceptions of how one's appearance is viewed by others, highlighting the heterogeneity of the instruments currently available and the lack of a measure specifically developed for this population. The use of BI instruments has also been reported in other neurological populations. In people with acquired brain injury, the Body Image Scale (BIS) has been used (Corallo et al. 2021; Lo Buono, Bonanno, et al. 2023). Conversely, in people with Parkinson's disease, the Appearance Schemas Inventory‐Revised (ASI‐R) has been used to investigate appearance‐related BI (Proietti et al. 2025).

Despite their psychometric robustness, none of these instruments has been specifically developed and validated to comprehensively assess BI in PwMS. This highlights the need for a population‐specific measure that adequately captures the BI experiences of people living with MS. The BIS is a patient‐reported outcome measure (PROM) originally developed for cancer patients (Hopwood et al. 2001) and has been shown to be a reliable instrument for detecting BI dissatisfaction. The original BIS has been translated into several languages, including Greek (Anagnostopoulos and Myrgianni 2009), Portuguese (Moreira et al. 2010), Korean (Khang et al. 2013), Dutch (van Verschuer et al. 2015), Spanish (Gómez‐Campelo et al. 2015), Turkish (Karayurt et al. 2015), Italian (Cheli et al. 2017), German (Hartung et al. 2023), Thai (Moonkaew and Polsook 2023), Hindi (Belani et al. 2025), Tamil and Telugu (Shunmugasundaram et al. 2022), and Malay (Song et al. 2026). It was mainly adopted in different oncological populations such as patients with advanced cancer, colorectal cancer patients, breast cancer patients, patients with an ostomy as a consequence of a cancer, or mixed cancer population (including breast, gynecological, gastro‐intestinal, genitourinary, head and neck, hematologic, and respiratory cancer) (Belani et al. 2025; Cheli et al. 2017; Hartung et al. 2023; Melissant et al. 2018; Song et al. 2026). This questionnaire was also adopted in non‐oncological populations; in particular, it was validated for patients with post‐coronary artery bypass graft (Moonkaew and Polsook 2023) and used in a population of PwMS (Lo Buono, Bonanno, et al. 2023). Overall, the BIS has shown satisfactory measurement properties across different populations and language versions, with evidence supporting good structural validity, internal consistency, and test–retest reliability. However, the evidence for some measurement properties remains limited (Melissant et al. 2018).

Among available instruments, the BIS was considered particularly suitable, given its prior use in PwMS (Lo Buono, Bonanno, et al. 2023). Importantly, the BIS is also available in Italian and has been psychometrically validated in Italian women with breast cancer (Annunziata et al. 2018; Cheli et al. 2017). Although other BI questionnaires have been translated and validated in Italian populations (Calugi et al. 2006; Marano et al. 2007; Marzola et al. 2022), these instruments mainly assess eating‐disorder‐related BI concerns and have not been specifically validated in PwMS. Therefore, the BIS was selected as a suitable existing measure to be adapted for MS population.

However, since the original instrument was designed to assess BI following breast cancer, where visible changes in appearance and body integrity are common, some items may not adequately capture the alterations in body perception and embodiment that occur in PwMS in the absence of overt physical disfigurement. Based on these premises, the present study aims to adapt and validate the original Italian version of the BIS for the MS population and to examine its psychometric properties.

## 2 Materials and Methods

### 2.1 Cross‐Population Adaptation of the BIS

The Italian version of BIS (Cheli et al. 2017) was adapted for PwMS following established guidelines for PROMs, with particular attention to conceptual equivalence, expert review, and patient involvement (Mokkink et al. 2010b; Patrick et al. 2011). A three‐phase process was undertaken. In the first phase, a multidisciplinary expert panel composed of two psychologists (J.P., A.S.) and one physiotherapist (V.G.) reviewed the original questionnaire. The panel discussed and modified selected items to ensure coherence with the clinical characteristics and lived experiences of MS, while preserving the original meaning and structure of the items whenever possible. This resulted in a first modified version, *mBIS v*1. The second phase involved assessing content validity through a panel of five MS healthcare professionals, including one psychologist (M.G.), one occupational therapist (E.G.), two rehabilitation physicians (S.M., M.M.B.), and one physiotherapist (M.B.), who evaluated item clarity and relevance. Based on the experts’ feedback, a new version of the questionnaire, *mBIS v2* was defined. In the third phase, a cognitive debriefing was conducted with five PwMS (two women and three men), according to ISPOR task force recommendations (Wild et al. 2005), to evaluate item clarity, comprehensibility, and relevance from PwMS perspective.

### 2.2 Study Design and Setting

This is a cross‐sectional observational study based on an online survey conducted by the research team of the Italian Multiple Sclerosis Foundation (FISM), Genoa, Italy.

The survey was created using Microsoft Forms and distributed through the mailing list of PwMS followed by the Rehabilitation Service of the Italian Multiple Sclerosis Society (AISM) in Genoa, Italy. Individuals included in the mailing list had previously provided consent to be contacted for participation in research projects. Participants completed an anonymous online questionnaire collecting socio‐demographic and clinical information, together with the study outcome measures.

### 2.3 Participants

Individuals were eligible to participate in the study if they had a confirmed diagnosis of MS according to revised McDonald criteria (Montalban et al. 2025) and were aged 18 years or older. The mailing list used for recruitment included only PwMS who were followed by the Rehabilitation Service of the Italian Multiple Sclerosis Society (AISM) in Genoa, Italy, and who met these eligibility criteria.

Before accessing the questions on the survey, participants had to provide their electronic informed consent to participate, after reading information about the study, in accordance with the revised Declaration of Helsinki, by clicking on the option “proceed with the questionnaire.” The study has been approved by the Regional Ethics Committee (1/2025 ‐ DB ID 14293).

Participants were asked to complete an anonymous online questionnaire combining socio‐demographic characteristics (age, sex, educational level, and job) and clinical characteristics (disability level as measured by a self‐Expanded Disability Status Scale [self‐EDSS] (Collins et al. 2016), disease duration, disease course, and drugs). At the end of the survey, participants were asked whether they would be interested in taking part in the subsequent phase of the study. To express their willingness to participate, participants were asked to provide their email address, which was subsequently used to contact them for the test–retest assessment.

### 2.4 Outcome Measures

Following PROMs and questionnaires related to BI, mood disorders, and interoceptive awareness were completed:

- *Modified‐Body Image Scale (mBIS)* is the adapted version of the BIS (Hopwood et al. 2001). It is a 10‐item questionnaire used to assess BI perception, adapted for PwMS. Each question can be rated with four options: “not at all” (Score 0), “a little” (Score 1), “quite a bit” (Score 2), and “very much” (Score3). The final score is the sum of the 10 items, ranging from 0 to 30, with a zero score representing the absence of symptoms or distress and highest scores representing increasing symptoms and distress or more BI concerns.
- *Hospital Anxiety and Depression Scale (HADS)* (Costantini et al. 1999) consists of seven questions for anxiety (HADS‐A score) and seven for depression (HADS‐D score). Possible total scores range from 0–21 for both anxiety and depression. Based on norms, 0–7 is normal, 8–10 is borderline abnormal, and 11–21 is abnormal. It has been translated and validated in the Italian population (Costantini et al. 1999) and in PwMS (Honarmand and Feinstein 2009).
- *Multidimensional Assessment of Interoceptive Awareness (MAIA)* (Mehling et al. 2012) is a 32‐item state‐trait questionnaire to measure multiple dimensions of interoception by self‐report, in which the participant has to rate “how often each statement applies to you generally in daily life,” with ordinal responses coded from 0 (“never”) to 5 (“always”). This questionnaire has been translated and validated in the Italian population (Calì et al. 2015).

### 2.5 Psychometric Properties of mBIS and Strategy of Analysis

Psychometric properties assessment followed COSMIN guidelines (Mokkink et al. 2010a, 2010b).

#### 2.5.1 Content Validity

Within validity domain, content validity was assessed using both qualitative and quantitative approaches, as described in phases 2 and 3 of the adaptation process (Section 2.1), by describing the agreement on item clarity and relevance among both the expert panel and PwMS.

A detailed description of the content validity process is provided in Supporting Information S2.

#### 2.5.2 Construct Validity

Construct validity was assessed through several steps. Regarding item‐level validity, for each item, we examined response frequencies, non‐null response prevalence, and Spearman correlations (given the ordinal nature of item scores) with total score (corrected by excluding the item in question to avoid the risk of inflating the correlation coefficient). Item‐level missing data were handled using available‐item when missingness was < 10%, and participant mean substitution otherwise.

Structural validity was assessed using confirmatory factor analysis (CFA) with the weighted least squares mean and variance adjusted (WLSMV) estimator, which is appropriate for ordinal indicators. The hypothesized one‐factor structure was tested. The suitability of the data for factor analysis was assessed using the Kaiser–Meyer–Olkin (KMO) measure of sampling adequacy and Bartlett's Test of Sphericity, computed from the polychoric correlation matrix.

The minimum sample size requirement was guided by previous literature recommendations for CFA rather than by an a priori Monte Carlo simulation. Specifically, the recommendations of Wolf et al. (2013) were considered for a one‐factor model with multiple indicators and standardized factor loadings around 0.50; our sample size exceeded the suggested minimum under these conservative conditions.

Model fit was evaluated using the scaled fit indices provided for the WLSMV estimator, including the Comparative Fit Index (CFI), Tucker–Lewis Index (TLI), Root Mean Square Error of Approximation (RMSEA), and Standardized Root Mean Square Residual (SRMR). The following cut‐off values were considered indicative of good fit: CFI and TLI ≥ 0.95 and SRMR ≤ 0.08.

Construct validity was also assessed based on two hypotheses: (1) we expected a moderate negative correlation between BI (mBIS) and interoception awareness as measured by the MAIA. This expectation was based on a growing body of research (Todd et al. 2019) suggesting that in the general population interoceptive processes contribute not only to physiological self‐regulation but also to body representation and self‐perception, which are central to BI. And (2) we expected a positive moderate correlation between BI (mBIS) and anxiety and depressive symptoms through the HADS, based on evidence suggesting that alteration in BI can lead to high depression (Di Cara et al. 2022) and that higher levels of body dissatisfaction are associated with higher levels of anxiety (McCormack et al. 2025). These hypotheses were examined using Spearman's rank correlations between the total scores of the mBIS and MAIA and the HADS anxiety and depression subscales. Normality was assessed using the Kolmogorov–Smirnov test.

#### 2.5.3 Internal Consistency

Internal consistency was primarily assessed using McDonald's omega, as it does not require the assumption of tau‐equivalence (Dunn et al. 2014). Cronbach's alpha (Cronbach 1951) was additionally calculated to facilitate comparison with previous validation studies of the instrument. Values above 0.70 were considered indicative of acceptable internal consistency reliability.

#### 2.5.4 Test–Retest Reliability

Test–retest reliability was assessed using two administrations of the questionnaire to the same participants, separated by a 7–14‐day interval.

Weighted kappa statistics with squared weights were used to assess test–retest reliability scores of single ordinal items; the corresponding 95% CIs were estimated using the bootstrap method (1000 replications). Kappa values indicate fair (0.21–0.40), moderate (0.41–0.60), substantial (0.61–0.80), and almost perfect agreement (0.81–1.00) (Landis and Koch 1977).

Intraclass correlation coefficient (ICC) (Shrout and Fleiss 1979; Weir 2005), absolute‐agreement, 2‐way mixed‐effects model with corresponding 95% CI, was used to assess the test–retest reliability of the total score (Koo and Li 2016). Values > 0.75 indicate good reliability (Koo and Li 2016). The minimum sample size required for test–retest reliability analysis was estimated using the R package *ICC.Sample.Size*, assuming 80% power, a one‐sided alpha level of 0.05, an expected ICC of 0.85, and a null hypothesis ICC of 0.50, corresponding to the threshold for moderate reliability (de Vet et al. 2006; Haley and Fragala‐Pinkham 2006; Koo and Li 2016).

#### 2.5.5 Measurement Error

Measurement error (Terwee et al. 2007) was assessed on total score by calculating the standard error of measurement (SEM) and the minimum detectable change (MDC) at a 90% confidence level, assuming normally distributed test–retest differences (Chen et al. 2020; Graber et al. 2025).

$$SEM=SD_{baseline}\;*\;\sqrt{1\;-\;ICC}$$

$$MDC=1.65\;*\;SEM\;*\;\sqrt{2}$$

In SEM calculations, SD is the standard deviation of the sample at baseline.

## 3 Results

### 3.1 Cross‐Population Adaptation of the BIS

A three‐phase process was adopted to adapt the Italian version of the BIS. During the first phase, the multidisciplinary expert panel discussed and modified selected items to ensure coherence with the clinical characteristics and lived experiences of PwMS, while preserving the original meaning and structure of the items whenever possible. This resulted in a first modified version, *mBIS v1*. The second phase involved a panel of five MS healthcare professionals for assessing content validity, in particular item clarity and relevance. Based on the experts’ feedback, several non‐conceptual adaptations were made to the Italian version of the BIS to ensure linguistic clarity, gender inclusiveness, and relevance to the target population, leading to a new version of the questionnaire, *mBIS v2*. Notable changes to the items included using gender‐inclusive language and removing references to treatment in favor of focusing on disease‐related effects. Throughout the questionnaire, no explicit reference to MS was included; instead, the generic term “disease” was retained to allow the instrument to be applicable across a wide range of populations with different diagnoses that may impact BI. For ease of comparison, Supporting Information S3 provides a table presenting the items of the original Italian version of the BIS alongside those of the mBIS. In the third phase, a cognitive debriefing was conducted with five PwMS (two women and three men) to evaluate item clarity, comprehensibility, and relevance from the patient perspective. Participants rated the comprehensibility of the items and the overall questionnaire. All items were deemed clear, and feedback indicated high understandability and relevance. No further modifications were necessary, establishing the final version of the mBIS for PwMS (see Supporting Information S1). In Supporting Information S2, we included a more detailed description of the adaptation process and results, including the modifications made to the items.

### 3.2 Participants

Based on previous literature recommendations for CFA, a minimum sample size of approximately 90 participants was considered adequate under conservative conditions (Wolf et al. 2013). The present study included 208 PwMS (age (SD): 50.9 (14.6); gender (F): 144; self‐EDSS (SD): 4 (1.96)), exceeding this recommendation. See Table 1 for socio‐demographic data, clinical characteristics, and questionnaire scores. All individuals completed the mBIS, with only one missing item across the entire dataset. The sample obtained a mean (SD) total score of the mBIS of 12.9 (9.0) (median [IQR] = 12 (15)).

**TABLE 1:** Sample sociodemographic data, clinical characteristics, and PROMs scores.

Variable	Mean (SD)	Frequency (%)
Age (y)	50.9 (14.6)	
Sex	—	F: 144 (69.2%)
		M: 64 (30.8)
Level of education	—	Primary: 27 (13%)
		Secondary: 102 (49%)
		Bachelor's degree: 21 (10.1%)
		Master's degree: 45 (21.6%)
		Doctoral degree: 13 (6.3%)
Job	—	Worker: 122 (58.6%)
		Unemployed: 15 (7.2%)
		Economically inactive: 13 (6.3)
		Retired: 26 (12.5%)
		Disability pension: 32 (15.4%)
Disease duration (y)	14.7 (11.0)	—
Self‐EDSS	4 (1.96)	—
Disease course	—	RR: 119 (57.2%)
		PP: 48 (23.1%)
		SP: 29 (13.9%)
		PR: 12 (5.8%)
DMT	—	Yes: 170 (81.7%)
		No: 38 (18.3%)
HADS‐A score	9.7 (2.3)	—
HADS‐D score	11.8 (2.1)	—
MAIA	83.7 (23.8)	—
mBIS score	12.9 (9.0)	

Abbreviations: DMT = disease modifying therapy; F = female; HADS‐A = Hospital Anxiety and Depression Scale‐Anxiety; HADS‐D = Hospital Anxiety and Depression Scale‐Depression; M = male; MAIA = Multidimensional Assessment of Interoceptive Awareness; mBIS = modified Body Image Scale; PP = primary progressive; PR = progressive relapsing; RR = relapsing‐remitting, Self‐EDSS = self‐Expanded Disability Status Scale; SP = secondary progressive.

### 3.3 Psychometric Properties of mBIS

#### 3.3.1 Content Validity

The content validity was assessed through the second and third phases of the cross‐population adaptation process (see Section 3.1).

#### 3.3.2 Construct Validity

Mean item scores were close to 1.5, corresponding to the midpoint of the response scale (Table 2).

**TABLE 2:** Descriptive statistics for individual mBIS items.

Item	Mean	SD	Median	IQR	freq. of missing	0	1	2	3	Non‐null response prevalence
mBIS_1	1.30	1.05	1	2	0	58	64	52	34	0.72
mBIS_2	1.25	1.16	1	2	0	77	45	44	42	0.63
mBIS_3	1.35	1.05	1	2	0	54	64	54	36	0.74
mBIS_4	1.25	1.17	1	2	0	79	42	44	43	0.62
mBIS_5	1.11	1.13	1	2	0	87	47	39	35	0.58
mBIS_6	1.40	1.23	1	3	0	74	35	41	58	0.64
mBIS_7	0.92	1.05	1	2	1	103	37	48	19	0.50
mBIS_8	1.42	1.07	1	1	0	50	65	49	44	0.76
mBIS_9	1.54	1.09	2	1	0	47	51	60	50	0.77
mBIS_10	1.41	1.18	1	2.5	0	66	42	48	52	0.68

Abbreviation: mBIS = modified Body Image Scale.

Item‐total score correlations were consistently high, all exceeding 0.70 and being statistically significant, indicating strong internal coherence among the items (Table 3).

**TABLE 3:** Spearman correlations between individual items and the corrected total score.

Item	Rho	*p*‐value
mBIS_1	0.72	< 0 .001
mBIS_2	0.79	< 0 .001
mBIS_3	0.73	< 0 .001
mBIS_4	0.77	< 0 .001
mBIS_5	0.76	< 0 .001
mBIS_6	0.78	< 0 .001
mBIS_7	0.70	< 0 .001
mBIS_8	0.72	< 0 .001
mBIS_9	0.81	< 0 .001
mBIS_10	0.75	< 0 .001

Abbreviation: mBIS = modified Body Image Scale.

Bartlett sphericity test was significant (*p* < 0.001) and the Kaiser–Meyer–Olkin statistic was 0.92, indicating excellent sampling adequacy for factor analysis (Kaiser 1974). A CFA was conducted on the 10‐item scale. The resulting factor loadings (Table 4) showed a strong association of all items with the latent construct, with standardized loadings ranging from 0.77 to 0.89. The one‐factor model showed strong comparative fit (CFI = 0.977; TLI = 0.968) and an acceptable SRMR (0.059). The chi‐square test was significant (*χ* ^2^ = 176.5, df = 33, *p* < 0.001), and the RMSEA remained elevated (0.145, 90% CI: 0.124–0.166). Considering the high factor loadings and the overall pattern of fit indices, the results supported a predominantly unidimensional structure. To improve model fit, two correlated residuals between semantically overlapping items (items 8–10 and items 3–9) were allowed.

**TABLE 4:** Results of one‐factor confirmatory factor analysis. Raw and standardized factor loadings, standard error, and associated *p*‐values are presented for each item.

Item	Estimate	Std. err	Std. loading	*p*‐value
mBIS_1	1.000	—	0.827	< 0 .001
mBIS_2	1.073	0.033	0.888	< 0 .001
mBIS_3	0.991	0.038	0.820	< 0 .001
mBIS_4	1.068	0.035	0.883	< 0 .001
mBIS_5	1.058	0.031	0.876	< 0 .001
mBIS_6	1.072	0.035	0.887	< 0 .001
mBIS_7	0.980	0.038	0.811	< 0 .001
mBIS_8	0.931	0.044	0.770	< 0 .001
mBIS_9	1.055	0.036	0.873	< 0 .001
mBIS_10	0.997	0.039	0.825	< 0 .001

Abbreviation: mBIS = modified Body Image Scale.

Hypothesis testing analysis confirmed our two hypotheses partially. Specifically, it revealed significant moderate correlations between the mBIS and the anxiety subscale (*r* = 0.52; *p* < 0.001 and a significant high correlation with the depression subscale (*r* = 0.67; *p* < 0.001) of the HADS. In contrast, a low negative correlation was observed between the mBIS and MAIA total scores (*r* = −0.24; *p* < 0.001).

#### 3.3.3 Internal Consistency

Internal consistency was excellent, with McDonald's omega = 0.963. Cronbach's alpha was additionally reported (*α* = 0.942) for comparability with previous studies (Youngstrom et al. 2018).

#### 3.3.4 Reliability and Measurement Error

Test–retest reliability was assessed on a sample of 26 PwMS (age (SD): 51.2 (11.3); gender (F): 20; self‐EDSS (SD): 3.2 (1.5)). The stability of individual items ranged from acceptable to good kappa (range 0.54–0.83). The total score exhibited very good reliability, with an ICC of 0.88 (95% CI: 0.76–0.95, *p* < 0.001) (Table 5). Overall, the scale showed satisfactory temporal stability at both the item and total‐score levels, demonstrating a good test–retest reliability of the questionnaire. SEM for total score was 2.88 and the MDC 6.73 (95% CI: 5.45–11.51).

**TABLE 5:** Test–Retest reliability assessed using the intraclass correlation coefficient (ICC) for total score and weighted kappa for the items. 95% confidence intervals (CIs) are reported in parentheses.

Item	Kappa	ICC	*p*‐value
mBIS_1	0.54 (0.19–0.82)	—	0.003
mBIS_2	0.76 (0.54–0.90)	—	0.000
mBIS_3	0.82 (0.67–0.91)	—	0.000
mBIS_4	0.60 (0.31–0.76)	—	0.002
mBIS_5	0.73 (0.45–0.89)	—	0.000
mBIS_6	0.83 (0.63–0.93)	—	0.000
mBIS_7	0.62 (0.30–0.81)	—	0.0014
mBIS_8	0.68 (0.43–0.83)	—	0.000
mBIS_9	0.73 (0.49–0.87)	—	0.000
mBIS_10	0.61 (0.40–0.75)	—	0.0014
mBIS_TOT	—	0.88 (0.76–0.95)	0.000

Abbreviation: mBIS = modified Body Image Scale.

## 4 Discussion

The aim of this study was to adapt the Italian version of the BIS to assess BI in PwMS and to validate the new version in a sample of PwMS. Given that BI is closely linked to psychological well‐being, self‐esteem, and overall quality of life, the availability of a validated PROM for PwMS represents an important step toward more comprehensive clinical assessment.

Regarding content validity, a three‐phase process involving both MS healthcare specialists and PwMS was conducted to define the mBIS. Changes to the items and instructions included using gender‐inclusive language and removing references to treatment or scars in favor of focusing on disease‐related aspects.

The psychometric analyses were conducted on a sample of 208 PwMS. Results highlighted the good structural validity of the mBIS, as part of construct validity. Specifically, the one‐factor structure was confirmed, in line with previous findings for both the original English version of the BIS (Hopwood et al. 2001) and the Italian version validated in people with cancer (Cheli et al. 2017). Moreover, the item‐total score correlations were consistently high, all exceeding 0.70. These values were overall higher than those reported for the Italian BIS, which ranged from 0.487 to 0.806 (Cheli et al. 2017).

Item 7 (“Did you avoid people because of the way you felt about your appearance?”) showed the highest frequency of zero responses, a lower item mean score, and the lowest item‐total score correlation (0.70, *p* < 0.001). These findings are consistent with those reported by Chieli et al. (2017), who observed an even lower mean score and item‐total correlation for this item. These results may suggest that, in different populations, such as women with cancer undergoing surgery and PwMS, BI perception has a limited impact on social avoidance and participation. In fact, although BI perception has been linked to social behaviors in clinical and non‐clinical populations, its direct impact on social avoidance and participation appears to be limited or mediated by factors such as fear of negative evaluation and social support, rather than representing a strong independent predictor across all groups (Finn et al. 2024).

Construct validity was also assessed by testing two hypotheses: (1) a moderate negative correlation between BI and interoceptive awareness, and (2) a positive moderate correlation between BI and anxiety and depressive symptoms. Regarding the first hypothesis, our results confirmed the direction of the association, although they only partially supported its magnitude. Specifically, low negative correlation between the BI (mBIS) and interoceptive awareness (MAIA) was observed. These findings are in line with previous research suggesting an association between the two constructs (Todd et al. 2019). In particular, lower levels of interoceptive awareness have been shown to be significantly associated with a more negative BI (Badoud and Tsakiris 2017). The magnitude of the association may be lower than that reported in the general population (Todd et al. 2019), as both interoception and BI perception can be altered by the underlying pathophysiological mechanisms of MS itself (Di Cara et al. 2019). Concerning the second hypothesis, BI was moderately correlated with anxiety (HADS‐A) and highly correlated with depression (HADS‐D). These findings are consistent with a recent review that summarized the evidence on the relationship between BI and anxiety and depression, demonstrating a positive association between these factors (McCormack et al. 2025). Furthermore, a systematic review in PwMS (McCormack et al. 2025) suggested that alterations in BI may contribute to the development or maintenance of anxiety and depressive symptoms. Due to the cross‐sectional design, causal relationships cannot be determined, and it remains unclear whether negative BI perception and anxiety and depressive symptoms, both common in MS (Boeschoten et al. 2017), influence each other in MS.

Internal consistency was good and comparable to that of the original Italian version of the BIS. Test–retest reliability was overall good, ranging from 0.54 to 0.83, and the reliability of the total score was consistently higher than that reported for the BIS (Cheli et al. 2017). The results highlighted an SEM of 2.88 and an MDC of 6.73, indicating that variations exceeding this threshold can be interpreted as real changes beyond measurement error. To date, no study has directly reported the SEM or the MDC for the BIS. However, a systematic review (Melissant et al. 2018) of the measurement properties of the BIS estimated these parameters based on reliability data reported by some studies. The estimated MDC values ranged from 4.7 to 11.1, while SEM values ranged from 1.7 to 4.0. However, the comparison with our findings is challenging due to differences in the target population and the use of the original BIS, which was developed for individuals with cancer, rather than the modified version adapted for PwMS.

The mean mBIS score observed in our sample (12.9) was slightly higher than the score reported in the sample of women with cancer after surgery (11.03), which was used to validate the original Italian version of the BIS (Cheli et al. 2017). This qualitative comparison—rather than a formal statistical test between the two samples—suggests that the impact of BI concerns can be present even in a neurological condition such as MS, which typically does not involve visible physical changes like surgical scars, highlighting that BI perception is relevant beyond conditions with overt bodily alterations. Furthermore, our sample was predominantly female (69%) and showed a moderate level of disability (mean self‐EDSS 4.0, SD 1.96), a profile broadly consistent with epidemiological data on MS. However, some protocol and sample characteristics should be considered when interpreting the comparison with the original Italian BIS validation sample. In our study, data were collected through an online survey, which requires access to and familiarity with digital devices; this modality may have selected participants with greater technological literacy and potentially different psychological profiles or coping strategies compared with samples recruited through in‐person and paper‐based administration, as in the original BIS validation. In addition, disease‐related factors such as EDSS may independently influence BI perception, consistent with previous findings (Di Cara et al. 2019) that confirmed that disability‐related changes in physical appearance can significantly affect the mental representation of BI. Taken together, these factors suggest that the comparison between samples should be interpreted with caution, as differences in mBIS scores may reflect not only the impact of the underlying condition but also sample composition and data collection modality. Future studies should specifically investigate which clinical and disease‐related factors may influence mBIS scores in PwMS.

Taken together, these findings highlight the importance of considering the impact of MS on BI in both psychological and rehabilitation programs. Although research on interventions specifically targeting BI in this population is still limited, preliminary evidence suggests that improvements may be achieved through physiotherapy and neurocognitive interventions (McCormack et al. 2025). Given the demonstrated associations between BI, anxiety, and depression, incorporating strategies aimed at supporting BI within psychological care programs may provide additional benefits for emotional well‐being in PwMS. Addressing BI concerns could therefore represent a valuable component of comprehensive, multidisciplinary care in MS.

Although the mBIS was adapted to assess BI in PwMS, it does not include specific references to MS in the items and instructions; instead, it refers only to “disease” in a general sense. This was an intentional design choice, aimed at creating questionnaire that could be applied across different clinical populations in which the disease may impact BI regardless of the presence of visible physical changes, such as surgical scars. Consequently, the questionnaire can be used in a wide range of conditions, including neurological disorders such as stroke (Davidson et al. 2025), acquired brain injury (Lo Buono, Corallo, et al. 2023), and Parkinson's disease (Gamarra et al. 2009).

### 4.1 Limitations

This study is not without limitations. One limitation is related to the content validity process, particularly to the third phase, in which only five PwMS were involved to evaluate item clarity, comprehensibility, and relevance from the patient perspective. Although involving five patients is considered sufficient for assessing content validity (Wild et al. 2005), this sample size cannot fully represent the heterogeneity of the MS population. Therefore, some perspectives or experiences of individuals with different sociodemographic or clinical characteristics may not have been captured. The cross‐sectional design prevents any conclusions about causality among BI, interoception, anxiety, and depression. Moreover, data collected via an online survey may have introduced selection bias. Participants with access to technology, higher motivation, or interest in the topic are more likely to have responded, potentially limiting the representativeness of the sample. In addition, the reliance on self‐reported measures introduces the possibility of reporting bias, including social desirability or misperception of symptoms, which could affect the accuracy of the observed associations. The lack of supervision during survey completion may have introduced uncontrolled variability, and missing or incomplete responses could further affect data quality. The study did not assess participants’ engagement in current or previous psychotherapeutic or psychological interventions that may substantially affect interoceptive processes, BI, and emotional regulation, which could have influenced the observed associations.

## 5 Conclusions

This study supports the validity and reliability of the mBIS for assessing BI in PwMS. The mBIS demonstrated good structural validity, internal consistency, and test–retest reliability, with a MDC of 6.73 (95% CI: 5.45–11.51) and a SEM of 2.88, as well as meaningful associations with interoceptive awareness, anxiety, and depressive symptoms.

The findings highlight that BI concerns are relevant in MS and may play an important role in psychological well‐being. The observed associations with anxiety and depression further emphasize the clinical relevance of assessing BI as part of a comprehensive evaluation of PwMS. The use of non‐disease‐specific wording represents a strength of the mBIS, allowing its potential application across different clinical populations in which disease‐related factors may affect BI. Overall, the mBIS appears to be a valid and versatile instrument that may facilitate both research and clinical practice by enabling the assessment of BI in PwMS and other neurological conditions. Future longitudinal and interventional studies are warranted to clarify the causal relationships between BI, interoception, and psychological symptoms, and to evaluate the effectiveness of targeted interventions aimed at improving BI within multidisciplinary care pathways.

## Author Contributions

**Erica Grange**: conceptualization, methodology, supervision, Writing – original draft, Writing – review and editing. **Alessia Susini**: data curation, investigation, writing – review and editing. **Giampaolo Brichetto**: supervision, writing – review and editing. **Federica Di Antonio**: data curation, formal analysis, methodology, writing – original draft, writing – review and editing. **Jessica Podda**: conceptualization, methodology, project administration, supervision, writing – original draft, writing – review and editing.

## Funding

The work was funded by the Italian Multiple Sclerosis Foundation through Progetto Ricerca Speciale 2021 e 2025.

## Ethics Statement

Participants have to provide their informed consent to participate after reading information about the study in accordance with the revised Declaration of Helsinki by clicking on the option “proceed with the questionnaire.” The study has been approved by the Regional Ethics Committee (1/2025 ‐ DB ID 14293).

## Conflicts of Interest

The authors declare no conflicts of interest.

## Supporting information




**Supplementary Material 1**. Modified BODY IMAGE SCALE (mBIS)

**Supplementary Material 2**. Three‐phase process for the cross‐population adaptation of the Italian version of the BIS

**Supplementary Material 3**. Items comparison between the original Italian version of the BIS and the mBIS

## Data Availability

Data are available upon reasonable request from the authors.
