# Supplementary material for: Assessing Body Image in Multiple Sclerosis: Adaptation and Validation of the Modified Body Image Scale
Source: Brain Behav. 2026 Oct 16;16(10):e71796. doi: 10.1002/brb3.71796 (PMC13673222; doi:10.1002/brb3.71796)
Supplement: Supplementary file 1 [file BRB3-16-e71796-s001.docx]

| Items | Original questionnaire (BIS) | Modified questionnaire (mBIS) |
| --- | --- | --- |
| 1 | Si è sentita imbarazzata/o per il suo aspetto fisico? | Si è sentita/o imbarazzata/o per il suo aspetto fisico? |
| 2 | Si è sentita/o meno attraente fisicamente a causa della malattia o del trattamento? | Si è sentita/o meno attraente fisicamente a causa della malattia? |
| 3 | Si è sentita insoddisfatta/o dal suo aspetto quando è vestita/o? | Si è sentita/o insoddisfatta/o dal suo aspetto quando è vestita/o? |
| 4 | Si è sentita/o meno femminile/virile a causa ella malattia o del trattamento? | Si è sentita/o meno femminile/virile a causa della malattia? |
| 5 | Ha avuto difficoltà nel guardare il suo corpo nudo? | Ha avuto difficoltà nel guardare il suo corpo nudo? |
| 6 | Si è sentita/o meno attraente sessualmente come risultato della malattia o del trattamento? | Si è sentita/o meno attraente sessualmente come risultato della malattia? |
| 7 | Ha evitato di incontrare persone a causa di come si sentiva in relazione al suo  aspetto? | Ha evitato di incontrare persone a causa di come si sentiva in relazione al suo aspetto? |
| 8 | Ha mai avuto la sensazione che il trattamento abbia lasciato il suo corpo meno integro? | Ha mai avuto la sensazione che la malattia abbia modificato il suo corpo? |
| 9 | Si è sentita/o insoddisfatta del suo corpo? | Si è sentita/o insoddisfatta/o dell’aspetto del suo corpo? |
| 10 | Si è sentita/o insoddisfatta/o dell’aspetto della sua cicatrice? | Si è sentita/o insoddisfatta/o di qualche aspetto visibile sul suo corpo causato dalla malattia (es. postura del corpo errata, atteggiamenti alterati di mani/piedi/braccia/gambe, perdita di massa muscolare, ausili, tutori, ecc…)? |

**Supplementary Material 3.** Items comparison between the original Italian version of the BIS and the mBIS

BIS=Body Image Scale; mBIS=modified-Body Image Scale
