# Supplementary material for: Assessing Body Image in Multiple Sclerosis: Adaptation and Validation of the Modified Body Image Scale
Source: Brain Behav. 2026 Oct 16;16(10):e71796. doi: 10.1002/brb3.71796 (PMC13673222; doi:10.1002/brb3.71796)
Supplement: Supplementary file 2 [file BRB3-16-e71796-s002.docx]

**Supplementary Material 2. Three-phase process for the cross-population adaptation of the Italian version of the BIS**

The Italian version of the Body Image Scale (BIS)^11^, originally developed for women with cancer, was adapted to PwMS following established recommendations for the content adaptation of patient-reported outcome measures, with particular attention to conceptual equivalence, expert review, and patient involvement^12,13^.

A three-phase process was followed. In the first phase, a multidisciplinary expert panel composed of two psychologists (JP, AS) and one physiotherapist (VG) reviewed the original Italian version of the questionnaire. The panel discussed and modified selected items to ensure coherence with the clinical characteristics and lived experience of MS, while preserving the original meaning and structure of the items whenever possible. This phase aimed to achieve conceptual relevance to the target condition with minimal necessary changes, in line with COSMIN recommendations^13^. At the end of this phase, a first version of the modified questionnaire was proposed (mBIS v1).

The second phase was dedicated to content validity assessment by MS healthcare professionals. The revised version of the questionnaire (mBIS v1) was subsequently submitted to a second panel of five healthcare professionals specialized in MS, including one psychologist (MG), one occupational therapist (EG), two rehabilitation Physician (SM, MMB), and one physiotherapist (MB). Each expert was asked to evaluate each item answering the following two questions: 1) “Are the statement and response options clear and understandable?” and 2) “Is the content of the statement relevant to the content of BI and to MS?”. For each question, experts could respond “yes,” “no,” or “partially,” and were invited to propose alternative wording when clarity or relevance was deemed insufficient. This procedure was conducted to formally assess content validity through expert judgment, as recommended by established guidelines for patient-reported outcome measures ^12^.

Regarding item clarity, items 1 to 4 were rated as clear by all five panelists. Items 6, 7, and 9 were judged as clear by four panelists, while one panelist rated each of these items as partially clear. Item 10 was rated as clear by three panelists, whereas two panelists judged it as partially clear. Item 8 was considered partially clear by four panelists and clear by one panelist. With respect to item relevance, items 2, 3, 7, 9, and 10 were rated as relevant by all panelists. Items 1, 4, and 6 were judged as relevant by four panelists, with one panelist rating each item as partially relevant. Item 8 was rated as partially relevant by two panelists and as relevant by the remaining three panellists.

Based on the experts’ feedback, several non-conceptual adaptations were made to the Italian version of the BIS to ensure linguistic clarity, gender inclusiveness, and relevance to the target population, leading to a new version of the questionnaire (mBIS v2).

To improve comprehensibility, gender-inclusive grammatical forms were explicitly introduced where required by the Italian language. Specifically, items 1 and 3 were modified by including both masculine and feminine verb forms (“sentita/o”), and item 9 by explicitly including both gender forms of the adjective (“insoddisfatta/o”).

To enhance content relevance for the target population, references to “treatment” were removed from items 2, 4, and 6, as BI concerns were primarily related to the disease itself rather than its treatment. These items were therefore reformulated to refer exclusively to disease-related effects.

Item 8 was further adapted by replacing the term “treatment” with “disease” and rephrased to more broadly capture perceived disease-related bodily changes, rather than the concept of bodily “wholeness,” which was considered less applicable. Finally, to ensure comprehensiveness and relevance, item 10 was substantially modified by removing the specific reference to surgical scars, which was not applicable to the target population. The item was reformulated to assess dissatisfaction with any visible disease-related bodily changes, and examples (e.g., altered posture, changes in limb positioning or movement, muscle mass loss, use of assistive devices or orthoses) were added to improve clarity and interpretability. Finally, a minor modification was made to the questionnaire instructions by removing references to “treatment” and shifting the focus to “disease,” in line with the changes applied to the individual items. Throughout the questionnaire, no explicit reference to MS was included; instead, the generic term “disease” was retained to allow the instrument to be applicable across a wide range of populations with different diagnoses that may impact BI.

The third phase consisted in a cognitive debriefing with PwMS. Specifically, the mBIS v2 was administered to five PwMS (two women and three men) to evaluate item clarity, comprehensibility, and relevance from the patient perspective. For each item, participants were asked whether “the statement and response options are clear and understandable,” with response options “yes,” “no,” or “partially.” In addition, participants rated their agreement on a 5-point Likert scale (1 = not at all, 5 = extremely) with the following statements: 1) The instructions and questions are easy to understand; 2) The length of the questionnaire (number of items, completion time) is appropriate; 3) The questions reflect difficulties that a person with MS may experience; 4) It is important to study this difficulty in people with MS; 5) The terms used in the questionnaire are clear.

This cognitive debriefing process was conducted to ensure that the adapted questionnaire was understandable, relevant, and acceptable to the target population, in accordance with recommended qualitative methods for evaluating content validity and item comprehension ^12,36^. All PwMS rated the 10 questionnaire items as clear and comprehensible. Regarding the five additional questions, all PwMS assigned the maximum score (5=strongly agree), except for one participant who rated all questions, except for one, with a score of 4 (4=agree), representing a minor variation in agreement. These findings indicate a high level of understandability, feasibility, relevance, importance, and clarity of both the individual items and the questionnaire as a whole.

No further modifications were made, and the final version of the modified-Body Image Scale (mBIS) adapted for PwMS was established (see Supplementary Material 1).
