# Supplementary material for: Assessing Body Image in Multiple Sclerosis: Adaptation and Validation of the Modified Body Image Scale
Source: Brain Behav. 2026 Oct 16;16(10):e71796. doi: 10.1002/brb3.71796 (PMC13673222; doi:10.1002/brb3.71796)
Supplement: Supplementary file 3 [file BRB3-16-e71796-s003.docx]

**Modified BODY IMAGE SCALE (mBIS)**

In questo questionario le verrà chiesto di descrivere come si sente rispetto al suo aspetto fisico e rispetto ad ogni cambiamento che possa essere derivato dalla malattia. La preghiamo di leggere attentamente ogni domanda e selezionare la casella che descrive al meglio come si è sentita/o **nell’ultima settimana** (Da "Mai" a "Molto").

Si prega di rispondere a tutte le domande.

|  |  | **MAI** | **POCO** | **MODERATAMENTE** | **MOLTO** |
| --- | --- | --- | --- | --- | --- |
| **1** | Si è sentita/o imbarazzata/o per il suo aspetto fisico? |  |  |  |  |
| **2** | Si è sentita/o meno attraente fisicamente a causa della malattia? |  |  |  |  |
| **3** | Si è sentita/o insoddisfatta/o dal suo aspetto quando è vestita/o? |  |  |  |  |
| **4** | Si è sentita/o meno femminile/virile a causa della malattia? |  |  |  |  |
| **5** | Ha avuto difficoltà nel guardare il suo corpo nudo? |  |  |  |  |
| **6** | Si è sentita/o meno attraente sessualmente come risultato della malattia? |  |  |  |  |
| **7** | Ha evitato di incontrare persone a causa di come si sentiva in relazione al suo aspetto? |  |  |  |  |
| **8** | Ha mai avuto la sensazione che la malattia abbia modificato il suo corpo? |  |  |  |  |
| **9** | Si è sentita/o insoddisfatta/o dell’aspetto del suo corpo? |  |  |  |  |
| **10** | Si è sentita/o insoddisfatta/o di qualche aspetto visibile sul suo corpo causato dalla malattia  (es. postura del corpo errata, atteggiamenti alterati di mani/piedi/braccia/gambe, perdita di massa muscolare, ausili, tutori, ecc…)? |  |  |  |  |

**Modified BODY IMAGE SCALE (mBIS)**

**Scoring**

Punteggio da attribuire a ciascuna risposta:

0 = mai

1 = poco

2 = moderatamente

3 = molto

I punteggi mancanti in uno o due items devono essere sostituiti dalla media degli altri items. Nel caso vi siano più di due punteggi mancanti il questionario è da considerarsi nullo.

Il punteggio totale è dato dalla somma algebrica di tutti gli items.

Il campione di riferimento della validazione della mBIS (Grange et. al) si riferisce a persone con sclerosi multipla.

L’adattamento della versione italiana della mBIS è stato realizzato da Erica Grange, Alessia Susini, Giampaolo Brichetto , Federica Di Antonio , Jessica Podda nell’articolo *“Assessing Body Image in Multiple Sclerosis: Adaptation and Validation of the modified Body Image Scale”*
